# PC_ali: a tool for improved multiple alignments and evolutionary inference based on a hybrid protein sequence and structure similarity score

**DOI:** 10.1093/bioinformatics/btad630

**Published:** 2023-10-17

**Authors:** Ugo Bastolla, David Abia, Oscar Piette

**Affiliations:** Centro de Biologia Molecular “Severo Ochoa” (CBMSO), CSIC-UAM Cantoblanco, 28049 Madrid, Spain; Bioinformatics Facility CBMSO, CSIC-UAM Cantoblanco, 28049 Madrid, Spain; Centro de Biologia Molecular “Severo Ochoa” (CBMSO), CSIC-UAM Cantoblanco, 28049 Madrid, Spain

## Abstract

**Motivation:**

Evolutionary inference depends crucially on the quality of multiple sequence alignments (MSA), which is problematic for distantly related proteins. Since protein structure is more conserved than sequence, it seems natural to use structure alignments for distant homologs. However, structure alignments may not be suitable for inferring evolutionary relationships.

**Results:**

Here we examined four protein similarity measures that depend on sequence and structure (fraction of aligned residues, sequence identity, fraction of superimposed residues, and contact overlap), finding that they are intimately correlated but none of them provides a complete and unbiased picture of conservation in proteins. Therefore, we propose the new hybrid protein sequence and structure similarity score PC_sim based on their main principal component. The corresponding divergence measure PC_div shows the strongest correlation with divergences obtained from individual similarities, suggesting that it infers accurate evolutionary divergences. We developed the program PC_ali that constructs protein MSAs either *de novo* or modifying an input MSA, using a similarity matrix based on PC_sim. The program constructs a starting MSA based on the maximal cliques of the graph of these PAs and it refines it through progressive alignments along the tree reconstructed with PC_div. Compared with eight state-of-the-art multiple structure or sequence alignment tools, PC_ali achieves higher or equal aligned fraction and structural scores, sequence identity higher than structure aligners although lower than sequence aligners, highest score PC_sim, and highest similarity with the MSAs produced by other tools and with the reference MSA Balibase.

**Availability and implementation:**

https://github.com/ugobas/PC_ali.

## 1 Introduction

The study of protein evolution relies heavily on the quality of multiple sequence alignments (MSA). However, it is known that distant alignments have low accuracy with consequent errors in evolutionary inference, which partly explains why current phylogenetic methods show poor performance when applied to distantly related proteins (Ogden and Rosenberg 2006, Lunter *et al.* 2008, Levy Karin *et al*. 2014, Mukarram *et al.* 2015). Different MSA programs applied to genomic scale datasets tend to produce qualitatively different conclusions (Wong *et al.* 2008), so that some researchers have advocated for the need of alignment-free approaches (Chan and Ragan 2013). The importance of alignments goes beyond evolutionary studies, as many bioinformatics methods rely on them. In particular, alignment quality has a strong influence on protein structure prediction both through homology modelling (Sali *et al.* 1995) and through correlated substitutions (Göbel *et al.* 1994, Weigt *et al.* 2009, Jumper *et al.* 2021), prediction of protein function (Radivojac *et al.* 2013), and molecular interactions (De Juan *et al.* 2013). The low accuracy of distant MSAs is particularly severe for superfamilies (Murzin *et al.* 1995, Orengo *et al.* 1997), which are the most distant groups of proteins whose common ancestry can be inferred based on sequence and structure similarity and that diversified their biological functions through long evolutionary histories.

Several approaches attempted to integrate structural information to improve MSA, using additional information such as predicted secondary structure (Jennings *et al.* 2001, Tong *et al.* 2015) or the statistical properties of gaps in structurally aligned proteins (Wrabl and Grishin 2004, Hijikata *et al.* 2011). These approaches are based on the observation that protein structure is more conserved than protein sequence (Rost 1997, Illergard *et al.* 2009, Pascual-García *et al.* 2010), so that structure similarity may still yield valuable information when sequence divergence is close to saturation. Consistently, it was found that structure-based alignments tend to be more accurate on benchmark databases, in particular for distantly related proteins and for buried residues; nevertheless, methods that combine sequence and structure information in general do not outperform structure-based methods (Carpentier and Chomilier 2019). Moreover, we found that structure alignment program tends to underestimate sequence identity (Pascual-García *et al.* 2019), questioning their usefulness for evolutionary studies.

## 2 Approach

Here, we consider diverse sequence and structure similarity measures. Each of them captures correlated but different aspects of protein evolution. Therefore, we derive the hybrid sequence and structure similarity measure PC_sim that captures all of them and we use it for improving an input MSA or for building an MSA *de novo* when structure information is available.

Sequence and structure divergence provide consistent evolutionary information since they are strongly correlated (Chothia and Lesk 1986). However, natural selection acts with different strength on them. The rate of structure divergence tends to be slower than sequence divergence when measured in comparable units (see Materials and methods), in particular for proteins that conserve the molecular function (Pascual-García *et al.* 2010), since mutations that conserve the protein structure but may affect other properties such as the folding stability or the metabolic cost of the protein are more frequently fixed than mutations that change the structure. This suggests that natural selection constrains protein structure more strongly than sequence. Conversely, proteins that change molecular function tend to evolve faster, in particular for structure divergence, which suggests that protein structure change is a target of positive selection (Pascual-García *et al.* 2019).

There are several measures of protein structure similarity and divergence, and we distinguish two main types. (i) Some similarities, such as the fraction of spatially superimposed residues, are computed after spatial superimposition, which depends on an optimized rotation matrix. A common criterion for determining the optimal rotation consists in maximizing the template-model (TM) score (Zhang and Skolnick 2004) (see Materials and methods) that trades off small root-mean-square deviation (RMSD) and large number of superimposed residues. The average protein coordinates in the native state allow predicting through the structure-based elastic network model (ENM) (Tirion 1996, Bastolla 2014a) native dynamical fluctuations that agree reasonably with experiments and correlate with observed large-scale functional motions (Tama and Sanejouand 2001). Therefore, we expect that proteins with high TM-score present similar native dynamics, as predicted through their ENM. (ii) The fraction of shared inter-residue contacts (contact overlap, CO; see Materials and methods) is a structure similarity measure that does not require any rotation. These contacts allow estimating the folding stability of the protein through simple contact-based models (Bastolla 2014b), and we expect that the CO correlates with the similarity of folding free energies. It is thought that protein dynamics is a target of selection more relevant than protein stability that evolves almost neutrally (Taverna and Goldstein 2002). Consistently with this expectation, we and coworkers observed that the TM-score decays more slowly than the CO in protein evolution and it is subject to stronger accelerations upon function change (Pascual-García *et al.* 2019), which is consistent with the above idea that protein dynamics is subject to stronger evolutionary pressure, both negative and positive, than protein stability.

Given the intricate sequence–structure–function relationship that characterizes proteins and the fact that different similarity measures capture only part of it, we studied the correlations between these measures. Different similarity measures provide consistent information, since they are strongly correlated. We found here that their main principal component (PC) represents more than 75% of the total variance, depending on the type of alignment. We thus propose that the main PC of protein similarity measures (PC_sim) yields a convenient description of the similarity between proteins that integrates sequence conservation, conservation of the local coordinates (related with native dynamics), and conservation of the contact matrix (related with folding stability).

The protein similarity measures allow inferring evolutionary divergence time, similarly to how sequence identity allows computing the Tajima-Nei (TN) diverence  (Tajima and Nei, 1984). These inferred divergences are often adopted to build the guide tree for progressive MSA, which has a strong influence on the final MSA and biases the phylogenetic relationships inferred through maximum likelihood methods (Levy Karin *et al.* 2014, Mukarram *et al.* 2015). We found that the divergence measure PC_div obtained from PC_sim achieves the highest correlation with the other divergence measures, which suggests that it provides a robust inference of divergence time and guide tree.

We then developed the MSA program PC_ali, which constructs MSAs based on PC_sim. In a first step, PC_ali constructs the pairwise alignments (PAs) that target PC_sim either *de novo* or by modifying the input MSAs. For comparison, PC_ali also constructs PAs that target the structure similarity measures TM-score and CO. We found that the PAs that target PC_sim yield the highest or second highest value of all similarity scores, and that they arguably provide better performances than alignments that target individual measures. In the second step, PC_ali builds the starting MSA associated with the maximal cliques of the graph of the PAs based on PC_sim. This MSA constitutes the starting point for iterative applications of the progressive multiple alignment algorithm that recurs the guide tree computed with PC_div adopting a similarity measure based on PC_sim.

We tested the resulting MSAs against a benchmark of seven state-of-the-art aligners based either on structure [Mammoth-mult (Lupyan *et al.* 2005) and TM-align-mult (Dong *et al.* 2018)] or on sequence [Decipher (Wright 2015), MAFFT (Katoh and Standley 2013), T-coffee (Notredame *et al.* 2000), Muscle (Edgar 2004), and Clustal (Sievers *et al.* 2011)] plus the manual alignments of Balibase http://www.lbgi.fr/balibase/BalibaseDownload/ built with sequence and structure information (Thompson *et al.* 1999). For all of the examined scores, we found that the MSAs produced by our program achieves either higher or not significantly different scores than the other programs, except for sequence identity, which is higher than for structure aligners but lower than for sequence aligners. They also provide the highest hybrid similarity PC_sim. Moreover, they present the highest average similarity with the MSAs produced by the other tools and with the Balibase MSA, which is considered a reference.

## 3 Materials and methods

### 3.1 Alignment algorithms

For benchmarking our MSA program PC_ali, we generated MSAs with five commonly used programs based on sequence similarity: Clustal-Omega (Sievers *et al.* 2011), MAFFT (Katoh and Standley 2013), MUSCLE (Edgar 2004), T-coffee (Notredame *et al.* 2000), and Decipher (Wright 2015), which also considers predicted secondary structure; two multiple structure alignments (MStAs) programs Mammoth-mult (Lupyan *et al.* 2005) and TM-align-mult (Dong *et al.* 2018) and the Balibase database of MSAs manually curated based on protein sequence and structure (Thompson *et al.* 1999). In all cases, we used default parameters.

### 3.2 Protein similarity measures

For each pair of proteins with known structure, we computed the following global similarity measures, either in sequence or in structure:

- *Fraction aligned (ali)*: Fraction of positions that are considered homologs (no gaps) with respect to the maximum length of the two proteins. This normalization penalizes insertions and deletions, which can produce functional or structural changes, although different length may also derive from different crystallization constructs.
- *Sequence identity (SI)*: Fraction of aligned positions that share the same amino acid (note that indels are not scored by SI).
- *TM-score (TM)*: Fraction of spatially superimposed aligned positions. While the RMSD measures structure divergence for fixed number of superimposed positions, it cannot be used when this number is variable since there is a trade-off between the length of the superimposition and its RMSD. To address this problem, Zhang and Skolnick (2004) introduced the TM-score, defined as
  (1) $$\text{TM}=\text{max}\left(\frac{1}{L}\sum_{i}\frac{1}{1+{\left(\frac{d_{i}}{d_{0}}\right)}^{2}}\right),$$
  where *L* is the number of aligned positions, *d_i_* is the distance between the two alpha carbons aligned at column *i* after optimal rotation, $d_{0}=1.24{\left(L-15\right)}^{1/3}-1.8$ is the *L*-dependent distance expected for structurally unrelated positions, and the optimal rotation matrix is determined self-consistently by iteratively maximizing the TM-score.
- *Contact overlap (CO)*: Fraction of shared contacts between two aligned protein structures. Different from the TM-score, the CO does not depend on any rotation matrix, and it is defined as
  (2) $$\text{CO}=\frac{\sum_{ij}C_{ij}C_{a(i)a(j)}^{\prime }}{\sum_{ij}C_{ij}\sum_{ij}C_{ij}^{\prime }},$$
  where *C_ij_* and $C_{ij}^{\prime }$ are the binary contact matrices of the two protein structures defined as 1 if any pair of heavy atoms of residues *i* and *j* is closer than 4.5 Å and 0 otherwise, *a*(*i*), *a*(*j*) are the residues of the second structure aligned to residues *i*, *j* of the first one (excluding gaps). The CO is normalized so that its maximum value is 1.
- *PC similarity (PC)*: The PC is the weighted combination of the four similarity measures described above. The weights were determined through the principal component analysis of the four similarity measures for all the superfamilies studied in this work. Using the MAFFT alignment to compute the similarity scores, we obtained:
  PC=(0.84ali+0.79SI+0.95TM+0.95CO)/3.53.

We use these weights in this work. Other alignment programs yielded similar weights, which are reported in Supplementary Table S1.

### 3.3 Evolutionary divergences

To each pairwise similarity measure, we associate an evolutionary divergence that estimates the time during which the two proteins diverged. For sequence identity, we adopt the TN divergence (Tajima and Nei 1984) that represents the maximum likelihood estimate of the divergence time under the Juke–Cantor (JC) model of molecular evolution in which sites are regarded as independent, all amino acids have the same stationary frequency and all pairs of different amino acids have the same exchangeability. This estimate is simple and parameter-free. For the structure similarity measures, we define divergences formally equivalent to the TN divergence. We postulate that they can estimate the divergence time for suitably defined models of structure evolution analogous to JC, as supported by their strong correlation with the TN divergence observed in previous works (Pascual-García *et al.* 2010, 2019).

- *TN divergence* (Tajima and Nei 1984) is computed from sequence identity (SI) as
  (3) $${\text{TN}}_{\text{div}}=-\ln\left(\frac{\text{SI}-{\text{SI}}_{0}}{1-{\text{SI}}_{0}}\right),$$

where ${\text{SI}}_{0}=0.05$ is the sequence identity expected for unrelated sequences.

- *TM divergence* computed from the TM-score (TM) as
  (4) $${\text{TM}}_{\text{div}}=-\ln\left(\frac{\text{TM}-{\text{TM}}_{0}}{1-{\text{TM}}_{0}}\right),$$

where ${\text{TM}}_{0}=0.167$ is the TM-score expected under convergent evolution (Zhang and Skolnick 2004), which is independent of *L* because the TM-score is carefully normalized.

- *Contact divergence* (Pascual-García *et al.* 2010), computed from the CO as
  (5) $$\text{CD}=-\ln\left(\frac{\text{CO}-q(L)}{1-q(L)}\right),$$

where $q(L)=0.39L^{-0.55}+6.64L^{-0.67}$ is the CO expected under convergent evolution, *L* is the number of aligned residues.

- *PC divergence*: It is a new hybrid sequence–structure divergence measure computed from the PC similarity, Equation (3), as
  (6) $$\begin{array}{l} {\text{PC}}_{\text{div}}=-\ln\left(\frac{\text{PC}-{\text{PC}}_{0}}{1-\text{PC}}\right)\\ {\text{PC}}_{0}=\frac{\left(0.84{\text{ali}}_{0}+0.79{\text{SI}}_{0}+0.95{\text{TM}}_{0}+0.95q(L)\right)}{0.84+0.79+0.95+0.95},\;{\text{ali}}_{0}=0.5. \end{array}$$

Note that divergences can be evaluated only for similarities larger than expected without homology ($\text{SI}>{\text{SI}}_{0},\;\text{TM}>{\text{TM}}_{0},\;\text{CO}>q(L),\;\text{PC}>{\text{PC}}_{0}$).

### 3.4 Protein superfamilies

We studied four protein domain superfamilies: Globins, Ploops, NADP, and Aldolases, selected as they are among the largest superfamilies in the SCOP and CATH databases (Murzin *et al.* 1995, Orengo *et al.* 1997). Proteins were parsed into globular domains in the SCOP database (Murzin *et al.* 1995), from which we obtained the average native coordinates of the selected domains.

For each superfamily, we clustered protein structures with Contact Divergence <2.5 as in Pascual-García *et al.* (2010), because it is not possible to obtain an MStA with Mammoth if the structural domains are too divergent. We obtained two clusters each for Ploops and Aldolases and one each for Globins and NADP, so that we ultimately studied six clusters. The distribution of the sequence and structure similarity measures for each of the four largest clusters is shown in Supplementary Fig. S1, from which one can see that sequence identity covers a broad range, with 26% of the pairs below 20% (designated as the twilight zone), 61% between 20% and 50%, and 12% above 50%. Since proteins may have different conformations, we also considered proteins with identical sequences within one point mutation and grouped their structures, computing the structure similarity between groups as the maximum across all of their conformations. The number of conformations and sequence groups in each cluster were the following: Aldolase C1: 38, 15; Aldolase C2: 23, 9; Globins C1: 397, 71; NADP C1: 161, 92; Ploop C1: 150, 73; Ploop C2: 45, 16.

### 3.5 Modified Pas

We compute the similarity and divergence scores described above for the starting alignments as well as four PAs modified through the use of structure information. In the first part of the work, we implemented fast modified alignments that are based on the starting alignment and do not require to score gaps, which is a critical point of all alignment methods.

The first modification that we studied is the secondary structure-based alignment (SS_ali). It is based on the idea that the sequence and the structure alignment have different aims: to infer homology and to identify structurally equivalent residues, respectively. Thus, they need not coincide everywhere. For instance, if a residue inside a secondary structure element (SSE) is deleted, the structure of the mutated protein will rearrange so to maintain the structural integrity and, in the structure alignment, the resulting gap will move to one of the two ends of the SSE. Our program detects such cases and moves the gap in the direction in which the displacement is smaller, it computes sequence identity and TM-score both for the starting and the modified alignment, and it selects the largest similarity. If the TM-score of the modified alignment is higher, the CO is also taken from the modified alignment.

Next, we construct modified PAs that target the TM-score (TM_ali), the CO (CO_ali), or the PC similarity (PC_ali) while modifying the input alignment as little as possible, through the following procedure: (i) for each residue we identify the nearest residue in the other protein as the one that maximizes the target score (CO, inter-residue distance or PC_sim), which depends on the input alignment and the optimal rotation matrix; (ii) we identify as neighbors two residues that present a double match, i.e. i1 is the nearest residue of i2 and i2 is the nearest residue of i1; (iii) we align neighbors that are aligned in the input alignment, obtaining frames; and (iv) proceeding from left to right, we align neighbors that are intermediate between frames. We iterate this procedure, calling new neighbors using the modified alignment. In this way, we obtain modified PAs that are similar to the input alignment and increase the target score without having to specify the gap penalty parameter.

### 3.6 Clique-based multiple alignment

We then obtained an MSA from the set of modified PAs through the following graph-based algorithm. (i) We transform the PAs into a graph where residues of the *n* proteins are nodes and links connect aligned residues. The maximum number of links per residue is *n*. If all PAs are consistent with an MSA, each column of the MSA corresponds to a maximal clique in the graph, i.e. a maximal set of fully interconnected residues. (ii) We rank all nodes based on the clustering coefficient of the PA graph, i.e. the number of pairs of neighbors of the node that are neighbor between themselves. (iii) We determine the maximal cliques of the graph for each residue *i* iteratively, following the ordering of the clustering coefficient in order to accelerate the computation, exploiting the list of neighbors and taking care to avoid repeated computations. The first clique is constituted by *i* and its first linked residue $l_{1}(i)$. At each step *s*, we add to all previous cliques the residue $l_{s}(i)$ linked to *i*. If this residue is linked to all residues in the clique it is added to it, otherwise a new clique is created with all residues that are linked to both *i* and $l_{s}(i)$. Crucially, to reduce the computation time at each step we keep only the 100 largest cliques. The ranking is very fast because the size can only take values from 2 to *n*. When the neighbors of *i* are exhausted we store the largest cliques. For each residue we store the maximum size and the sum of the sizes of its cliques, and exclude from future computation residues for which any of them is larger than n/2. This precaution provides a good compromise between completeness and computational efficiency. (iv) We assemble the cliques that are reciprocally consistent, i.e. they do not violate sequential order, starting from the largest one. (v) Residues that do not belong to any maximal clique are assigned to the clique most connected to them if this is consistent with all other pre-existing cliques. Otherwise, unassigned residues seed a new column. (vi) We reconstruct the MSA from the set of all ordered columns (maximal cliques) and we store it for subsequent use.

### 3.7 Progressive multiple alignment

For improving the quality of the MSA, PC_ali iteratively applies a progressive multiple alignment algorithm that targets PC_sim. Based on the previous MSA, it optimally superimposes all the protein structures maximizing the sum of the TM-scores of the aligned residues, it computes the PC_div divergence measures for all pairs of proteins, and it uses PC_div for constructing a guide tree through the average linkage algorithm (we also explored the neighbor joining algorithm but obtained slightly poorer results, contrary to our expectation). All these tasks are internally programed in PC_ali.

At each step of the clustering algorithm, PC_ali constructs the similarity matrix of two clusters of sister proteins based on PC_sim, using the previous alignment for estimating the CO and for superimposing the structures, it uses the matrix Blosum62 for scoring sequence similarity and it also scores aligned pairs of residues. Subsequently, it aligns the two clusters with the Smith–Waterman algorithm (Smith and Waterman 1981) that we modified from the program ProFit (Martin, A.C.R., http://www.bioinf.org.uk/software/profit/). PC_ali selects the MSA with the largest average PC_sim score. It performs up to seven progressive MSA steps, but it can stop earlier if the scores converge or cycle. The computations are fast, and the progressive steps take longer than the clique-based MSA.

### 3.8 Initial PAs

PC_ali can work in two modes: either it reads an input MSA and uses it for obtaining the starting PAs (option -ali) or it constructs the PAs *de novo* (option -seq) with a scoring matrix inspired to PC_sim that considers secondary structure identity instead of TM-score and contact vector similarity (i.e. the similarities of the sequence distances |i−j| between residues in contact) instead of CO, in such a way that it does not need a starting PA.

### 3.9 Comparison of MSAs

To assess the MSA, we downloaded the Balibase set of structure-curated multiple alignments (Thompson *et al.* 1999). For each MSA, we only considered sequences that are associated with a protein structure in the PDB (Berman *et al.* 2000). We assessed the similarity between pairs of alignments through the sum of pairs score (Thompson *et al.* 1999) that sums the pairs of residues that are aligned in both alignments. When computing average scores without assuming a reference alignment, we adopted a symmetric version of the score, normalizing the sum through the geometric mean of the sum of aligned pairs in the two alignments, which also penalizes overalignments. We also considered the column overlap score, which is the fraction of identical residues that appear in corresponding columns of two MSAs. This score is similar to the column score of Balibase but it is more robust since it also scores cases in which two columns are not completely identical. However, it depends heavily on the number of gaps in the column and we consider it a less robust score than the sum of pairs score.

### 3.10 Output files

For each pair of proteins, the program PC_ali outputs for posterior analysis five similarity measures (ali, SS, TM, CO, and PC) and the corresponding divergences for the required PAs (input and PAs that target SS, TM, CO, and PC) and for the final MSA. The pairwise scores are printed both for all examined structures (.sim and .div) and for the representative structures of each cluster of identical sequences (.prot.sim and .prot.div). The program then prints the MSA in fasta format (.PCAli.fas), the MSA of secondary structures (.PCAli_ss.msa), the neighbor joining tree constructed with PC_div (.PCAli.tree) and the PDB file with optimally superimposed structures (.PCAli.pdb). The file .id presents an analysis of conservation patterns that we will present elsewhere, and the file _summary.dat reports the average scores obtained with the distinct pairwise and multiple alignments that are constructed.

## 4 Results

### 4.1 Correlations between similarity measures

**Figure 1.**
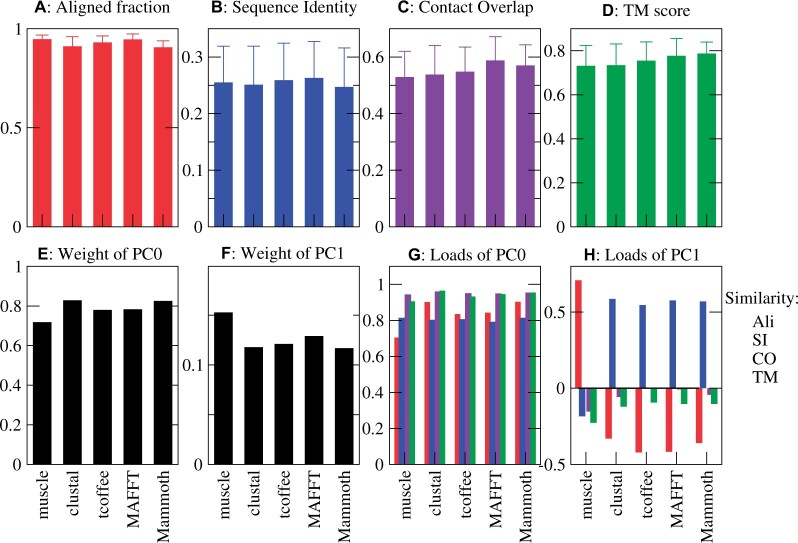
Similarity scores obtained by different alignment algorithms (A–D) averaged over all alignments of the four superfamilies, and their principal components (E–H). Error bars represent standard deviations.

In this work we consider four protein similarity measures: fraction of aligned residues (ali), fraction of aligned residues that are identical (SI), fraction of spatially superimposed residues (TM-score), and fraction of shared contacts (CO) (see Materials and methods). Figure 1A–D shows the similarity scores obtained with four sequence alignment programs [Clustal (Sievers *et al.* 2011), MAFFT (Katoh and Standley 2013), MUSCLE (Edgar 2004), or T-coffee (Notredame *et al.* 2000)] and one structure alignment program [Mammoth (Lupyan *et al.* 2005)] averaged over all alignments of four superfamilies. As expected, the sequence alignment programs attain higher sequence similarity scores and lower structure similarity scores than Mammoth (see Fig. 1), demonstrating that targeting different similarity scores biases the final results. Clustal and Mammoth tend to align fewer residues than the other programs (Fig. 1A). Mammoth obtained the highest TM-scores of all programs (Fig. 1D), which is not surprising since it targets a score similar to the TM-score. MAFFT achieved the second highest TM-score and the highest CO, even higher than the MStA program Mammoth (Fig. 1C).

As expected, all similarity measures are strongly correlated: two proteins whose alignment presents few gaps also present large sequence identity, large number of spatially superimposed residues, and large fraction of shared contacts. PC analysis shows that the main PC, which we call PC0, accounts for more than 75% of the total variance (Fig. 1E). All examined alignment programs yield similar values of the weight of PC0, with the MStA program Mammoth yielding the largest value. All similarity measures contribute positively to PC0 (Fig. 1G), and the measures that contribute most are the two structure similarity measures, which are strongly correlated between each other, while SI contributes least, except for Muscle. We interpret PC0 as an integrated measure of evolutionary relatedness, since it is large for pairs of proteins that are strongly related under the point of view of both sequence and structure. Therefore, we adopted PC0 as a new similarity measure, PC_sim, which integrates sequence and structure conservation. In this way, we weight more the scores that are more correlated and mutually support each other, which would produce similar alignments. We tested this choice by assessing alignments that target PC_sim, as reported below.

The PC loads are remarkably robust to the input MSA: their range is 0.79−0.81 (SI), 0.94−0.96 (CO), 0.91−0.97 (TM), with largest variation 0.71−0.90 for the load of the aligned fraction (see Supplementary Table S1). The loads determined with only one superfamily are very similar to those obtained with the full dataset (see Supplementary Fig. S2). One might expect that the loads vary with the sequence identity, but the subsets of very distantly related pairs (SI < 0.2) and intermediate ones (0.2<SI < 0.5) provide essentially the same loads, whereas for the few closely related pairs (SI > 0.5, 12% of pairs) the SI has a small load (see Supplementary Fig. S2) because it is weakly correlated with structure similarity and aligned fraction, possibly due to proteins with same sequence and different conformations. These results show that we can define the hybrid score PC_sim in a robust way.

The second PC (PC1) accounts for most of the remaining variance, but its weight is much smaller (Fig. 1F). It is contributed by the ali measure and the SI measure with opposite signs, while the structure similarities yield small contributions (Fig. 1H). This means that aligned proteins with large PC1 score have larger fraction of identical amino acids and fewer aligned residues, i.e. more gaps. This may suggest that PC1 arises from the tendency of alignment programs to overfit the sequence identity at the expense of placing gaps and slightly reducing the structure similarity. However, this interpretation is questioned by the fact that we observe PC1 also for alignments produced by the structure alignment program Mammoth that does not score sequence identity, although with a small weight. An alternative interpretation is that pairs with large PC1 are domains with different size, either because they were crystallized from different constructs or because they were differently parsed in the SCOP database. Among the sequence alignment programs, the lowest weight of PC1 is attained by Clustal, followed by T-coffee, MAFFT and then MUSCLE, for which it is largest.

### 4.2 Structure-guided PAs

We start presenting four structure-guided pairwise modifications of input MSAs. One of them is based on secondary structure (SS_ali), and the remaining three target structure similarity scores: TM_ali targets the TM-score, CO_ali targets the CO, and PC_ali targets the hybrid sequence and structure similarity score PC_sim.

**Figure 2.**
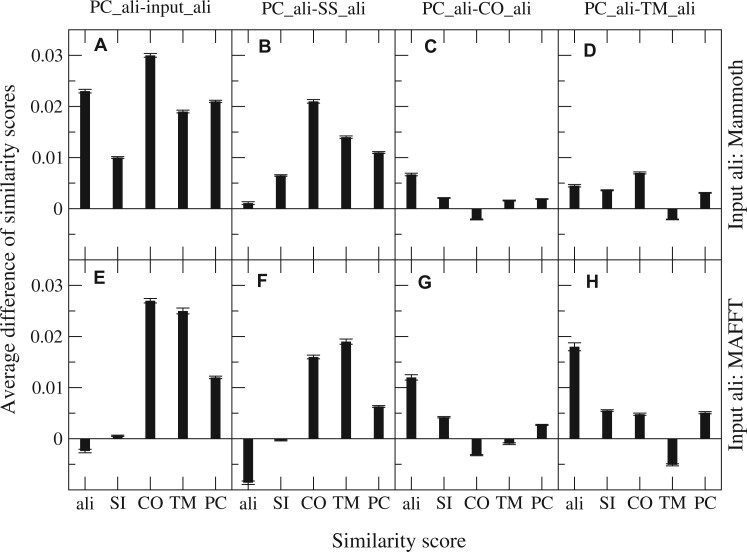
Difference between the similarity scores obtained through the pairwise PC_ali alignment and the input alignment (A, E) and three additional modified alignments (B, F: SS_ali; C, G: TM_ali; D, H: CO_ali), averaged over all the alignments of the four superfamilies. The examined scores are the aligned fracion (ali), the sequence identity (SI), the fraction of spatially superimposed residues (TM), the contact overlap (CO), and the hybrid PC_sim that integrates all of them (PC). The upper plots show the case in which the starting alignment is Mammoth, in the lower plots it is MAFFT. The error bars indicate the standard error of the mean difference.

Figure 2 shows the average differences in five similarity measures (ali, SI, TM, CO, and PC_sim) between the pairwise PC_ali and the input alignment or the other three modified PAs. One can see that the targeted score is always highest in the alignment that targets it compared to other alignments. This improvement happens at the expense of the other scores. The situation is different for PC_ali, which obtains the first or second highest score for all similarity measures, both sequence and structure, except a small decrease in the aligned fraction when the input alignment is MAFFT. Globally, PC_ali was the modification with the highest average improvement of the similarity measures.

When we used as input the MStA obtained through Mammoth, the PC_ali correction increases all the sequence and structure similarities (Fig. 2A), which suggests that the alignment quality overall improves. Of note, a recent paper reported that hybrid sequence–structure alignment methods performed worse than the Mammoth program (Carpentier and Chomilier 2019), which is not the case for the approach based on PC_ali. With the input MSA constructed with MAFFT, PC_ali improves all scores except the aligned fraction (Fig. 2E). We found qualitatively similar results with the MSAs constructed by other programs (see Supplementary Fig. S3). Interestingly, the scores obtained with PC_ali are more robust with respect to the input MSA than the scores obtained with the input MSA itself. In particular, using MAFFT or Mammoth as input MSA does not have a significant influence on the score PC_sim (see Supplementary Fig. S3).

The modified alignment SS_ali moves the gaps contained inside any SSE toward the closest end of the SSE. This is motivated by the idea that gaps inside a SSE might happen in evolution, so that the sequence alignment correctly infers homology, but the structure reorganizes so that the structural correspondence is different from the one dictated by the sequence alignment, i.e. sequence and structure alignment do not need to coincide. Accordingly, when we compute the average similarity scores we consider the higher between the SI score of the starting and the modified alignment, and the higher between the two TM-scores. Not surprisingly, this procedure increases all similarity scores in Fig. 2B and F. To test our interpretation, we consider the effect of SS_ali on the similarity scores without selecting the higher score. If SS_ali is capturing gaps inside SSE, we expect that the SI tends to decrease when the structure similarity scores TM and CO increase. Nevertheless, contrary to our interpretation, we found that in most cases SS_ali decreases the similarity scores SI, TM, and CO, both with respect of the sequence aligner MAFFT and with respect to the structure aligner Mammoth, i.e. modifications that improve the similarity are less frequent (Supplementary Fig. S4A). Moreover, sequence identity and structure similarity tend to increase or decrease together (see Supplementary Fig. S4B), which suggests that SS_ali is either correcting alignment errors through the use of secondary structure information or creating mistakes, instead of dealing with genuine cases of indels inside SSE that motivated it.

### 4.3 Divergence measures

The results presented above and other ones that we will present elsewhere suggest the existence of compensatory changes, particularly strong for closely related protein pairs, that make difficult to disentangle the evolutionary history of a protein superfamily in terms of only one similarity measure such as sequence identity, 3D superimposition or CO. These observations support our proposal to adopt the hybrid measure PC_sim that integrates various aspects of protein similarity. We now assess whether the new similarity measure can improve our ability to infer protein divergence.

From the comparison of the aligned sequences, we can infer the time past since the divergence of the two proteins using simple substitution models. This estimated divergence is often used to construct the guide tree for the processive multiple alignment algorithm and its accuracy has an important influence on the final results. The inferred divergence can be computed with simple formulas such as the TN divergence (Tajima and Nei 1984). Adopting the TN formula, we estimate divergence times using structure similarities. Although the analogy is only formal, we expect that these measures may be also derived from simple probabilistic models of protein structure evolution.

We compute the correlations between divergence measures through a linear model with an offset, D2=aD1+b. In principle the offset *b* should vanish, because the divergence *D*2 should vanish for D1=0. However, protein structures may differ even for identical sequences due to the presence of conformational changes or the influence of different experimental condition on the structure determination, so that in practice the offset *b* is never zero for structure divergence. We try to reduce the influence of conformational changes by considering the maximum structural similarity over all conformations of the same protein.

Since all divergence measures aim at inferring the same quantity, we assess their quality by measuring the average correlation coefficient with the other divergences, since the divergence measure with highest average correlation can estimate the other measures most reliably. We show in Supplementary Fig. S5 that the highest correlation (0.92 for alignments derived from MStA) is attained by the divergence of PC_sim (PC_div), Equation (6), which is therefore expected to provide the best inference of the divergence time among all divergence measures that we examined. On the contrary, the sequence-based TN divergence (Equation 3) has the lowest average correlation, followed by the divergences of the structural scores [TM-score (Equation 4) and CO (Equation 5)].

### 4.4 Multiple sequence alignments

As described in the Materials and methods section, we transform the PAs that target PC_sim into a graph and we determine its maximal cliques, from which we generate an MSA that we improve through subsequent progressive alignment steps. We finally select the MSA with largest PC_sim.

We assess these MSAs against eight multiple alignment programs on the Balibase set of MSAs (Thompson *et al.* 1999), retaining only sequences with available structure in the PDB. We omitted cases in which there were discrepancies between the Balibase and the PDB sequences, either because Balibase omits residues whose index in the PDB presents an insertion code (8% of the PDB sequences in Balibase) or because it includes more than one chain when the order of the chains in the PDB file is distinct from the alphabetic order. In some cases, we could save these sets by realigning the Balibase and PDB sequences.

**Figure 3.**
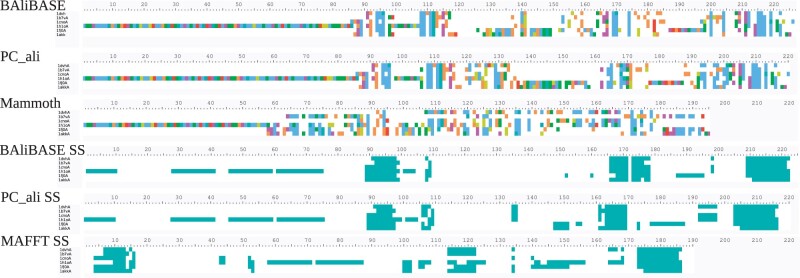
MSAs produced by Balibase (first and fourth row), PC_ali (second and fifth), Mammoth (third), and MAFFT (sixth row) for the Balibase set BB30027. We show either sequences (three top rows) or secondary structure elements (three bottom rows).

We show for illustration in Fig. 3 the MSAs produced by PC_ali, Balibase, Mammoth, and MAFFT for the Balibase set BB30027, which is short enough for visualizing the complete alignment. We present either sequences or SSEs. One can see that the PC_ali MSAs resemble the Balibase MSA more than those produced by MAFFT or Mammoth, it presents well-defined blocks and higher sequence or secondary structure identity comparable to the one of Balibase.

**Figure 4.**
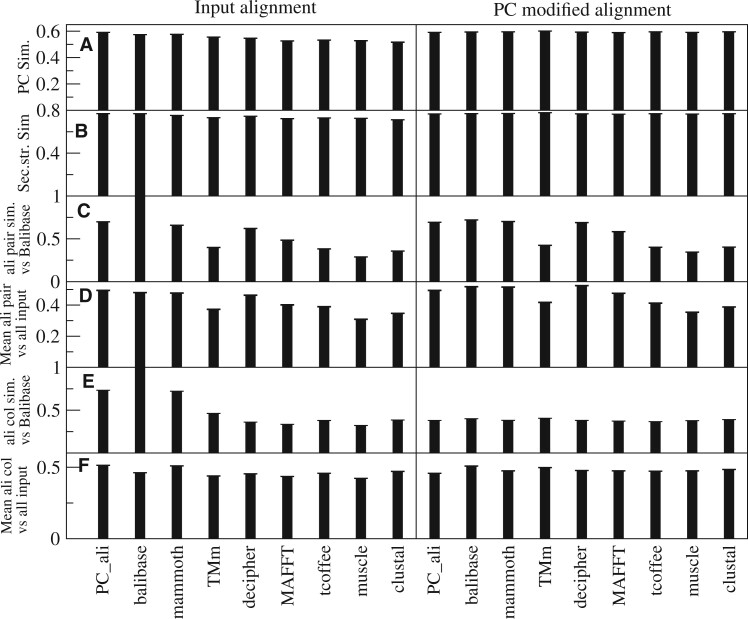
Scores of the MSA programs averaged over all Balibase MSAs. Error bars represent standard deviations. Left: Original MSA. Right: PC_ali modified MSA. (A) Hybrid sequence and structure PC_sim score. (B) Secondary structure similarity score. (C, E) Similarity with the Balibase reference alignment (C: sum of pairs, E: column overlap). (D, F) Average similarity with all other input MSAs, excluding the PC_ali modified MSAs (D: sum of pairs, F: column overlap).

**Figure 5.**
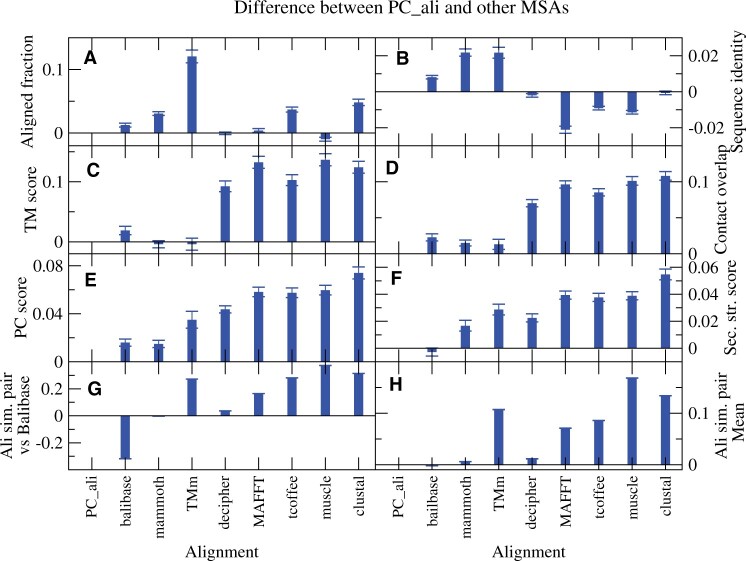
Average difference between PC_ali and other MSA programs. The error bars represent the standard error of the mean and allow to visually assess the significance. Only the input MSAs are shown. (A) Aligned fraction. (B) Sequence identity. (C) TM-score. (D) Contact overlap. (E) PC_sim. (F) Secondary structure identity. (G) Sum of pairs similarity with the Balibase reference MSA. (H) Average sum of pairs similarity with all other input MSAs, excluding the PC_ali modified MSAs.

The average scores of all nine methods and their PC_sim-corrected modifications are shown in Fig. 4, where the error bars represent the standard deviation. We represent the differences between PC_ali and the other scores in Fig. 5. The error bars represent the standard error of the mean (SEM). Since the mean of the difference divided by the SEM follows the student-*t* statistics, the error bars allow to visually assess the significance of the difference in the frequent cases in which the SEM is much smaller than the distance from zero. Not surprisingly, PC_ali achieves the largest PC_sim score (Figs 4A and 5E). It also achieves the second largest secondary structure score (Figs 4B and 5F), not significantly different from Balibase, although it is not scored by PC_sim.

PC_ali produces the MSAs most similar to those of Balibase, according both to the sum of pair score (Figs 4C and 5G, where PC_ali does not differ significantly from Mammoth) and to the column overlap score (Fig. 4E), and also according to the column score defined by Balibase (not shown). We obtain similar results for the average similarity with the other MSAs, excluding of course the PC_ali-modified MSAs that are very similar to each other, where PC_ali is the most central alignment (Fig. 4D and F).

Of course the similarity between MSAs depends on their sequence identity, as shown in Supplementary Fig. S6. At identity >50% PC_ali, Balibase Mammoth, and Decipher, which appear as the best methods, yield almost identical alignments (>95% similarity). In contrast, in the twilight zone <20% PC_ali is the most central, being most similar to Balibase by 60% (Supplementary Fig. S6A) while Mammoth is the most similar to PC_ali by 65% (Supplementary Fig. S6B), which also supports the quality of the PC_ali MSAs.

Concerning individual scores, PC_ali aligns more than all other methods (Fig. 5A), excluding Muscle that aligns slightly more and Decipher that presents no significant differences. In contrast, TM-align aligns much less, and Clustal, T-coffee, and Mammoth align significantly less. PC_ali achieves higher sequence identity than the structure aligners, including Balibase, but lower than the sequence aligners MAFFT, T-coffee, and Muscle, with similar scores as Decipher and clustal (Fig. 5B). These results evidence advantages of PC_ali with respect to structure aligners. PC_ali achieves TM-score comparable to Mammoth and TM-align, slightly higher than Balibase and much higher than all of the sequence aligners, of which Decipher and T-coffee appear as the best ones (Fig. 5C). PC_ali achieves higher CO than all other methods, including the structure aligners, although the difference with TM-align is not significant (Fig. 5D). PC_ali achieves the highest PC_sim hybrid score (Fig. 5E), which is not surprising because it optimizes this score. It is less obvious that it achieves the second-best secondary structure similarity score after Balibase, which is not significantly higher (Fig. 5F), because this score is not considered by PC_ali except in the initial pairwise step and it is considered by Balibase.

Finally, when analyzing the PC_ali-modified MSAs, we see that they all perform very similarly irrespective of the MSA from which they are derived (right part of Fig. 4). Most scores improve in the comparison between the input MSA and the modified MSA (Supplementary Fig. S7), except sequence identity for input MSAs that target sequence similarity, i.e. MAFFT, T-coffee, and Muscle (Supplementary Fig. S7B), similarity with Balibase when the input MSA is Balibase itself (Supplementary Fig. S7H and J), and column overlap similarity with Balibase and with other MSAs when the input MSA is PC_ali (Supplementary Fig. S7I and J). PC_ali is the input alignment that is least modified after targeting PC_sim and most of its scores are not significantly different (Supplementary Fig. S7), which shows that it is rather robust.

## 5 Discussion

Here we addressed the influence of the similarity score that is targeted by an alignment program, either sequence similarity or structure similarity measured either in terms of superimposed atomic coordinates [as in Mammoth (Lupyan *et al.* 2005) or TM-align (Zhang and Skolnick 2005)] or in terms of inter-residue contacts that are independent of rotations [as in the Dali program (Holm and Sander 1993)]. Different similarity scores are expected to be correlated if similarity is inversely correlated with evolutionary divergence, so that different criteria should identify aligned positions in a consistent way. However, the correlations between similarity measures are not perfect, and they may produce systematically different evolutionary inferences, since the adopted similarity measure has a strong influence on the resulting inferred homology.

In order to get more insight on the agreement and disagreement between different protein similarity measures, we analyzed four large superfamilies of homologous proteins with known structures that have diverged in sequence, structure, and function throughout a long evolutionary story (Murzin *et al.* 1995, Orengo *et al.* 1997): Globins, Aldolases, P-loop, and NADPH. Our analysis supports the idea that different properties tend to give consistent information, but not exactly interchangeable, since compensatory mechanisms reduce the correlation between similarity measures: for instance, contact conservation may be achieved through compensatory changes of the coordinates of the residues in contact. Therefore, we expect that no individual similarity measure can give an unbiased description of protein evolution, and it is useful to combine different measures. In particular, we confirmed that the global conservation scores of different properties are correlated, and we exploited these correlations for constructing a new integrated similarity score based on the main PC of sequence and structure similarity measures (see Fig. 1), which we called “PC_sim,” and which can exploit the synergies between different measures.

We then constructed three new PAs that modify an input MSA produced by sequence or structure alignment program by targeting three different similarity measures: spatial proximity after superimposition measured by the TM-score (Zhang and Skolnick 2004) (Equation 1), superimposition-independent similarity measured by the CO (Equation 2), and the hybrid sequence and structure similarity measure PC_sim. We developed an algorithm that identifies structural “neighbors” through double best match and produces new PAs that minimally modify the input MSA without having to score gaps. The similarity targeted by the alignment algorithm has a systematic influence on the resulting scores (see Fig. 2). Targeting structure similarity with input MSA derived from MAFFT tends to decrease the sequence identity, except with SS_ali and with PC_ali that targets PC_sim, which considers sequence identity. The purely structural modifications TM_ali and CO_ali increase the TM-score and the CO at the expense of sequence similarity and aligned fraction, with no increase of PC_sim in the case of TM_ali, which suggests that TM_ali may be overfitting the TM. On the other hand, the alignment PC_ali that targets PC_sim improves or maintains all similarity measures, and arguably it has the best performances. Thus, our results suggest that the hybrid sequence and structure alignment method based on PC_sim can produce high-quality alignments.

We then tested whether PC_sim improves the inference of the evolutionary divergence time. We adopted simple estimates of the evolutionary divergence formally analogous to the TN divergence measure obtained from sequence identity and often used in evolutionary studies (Tajima and Nei 1984): the contact divergence (Pascual-García *et al.* 2010) and the TM-score (Pascual-García *et al.* 2019) divergence and the hybrid sequence and structure measure PC_div, based on PC_sim. These four divergence measures are strongly correlated with each other, which supports their ability to infer the evolutionary time. We expect that the divergence measure most strongly correlated with the other ones provides robust inference of the evolutionary time. We found that PC_div shows the strongest correlations (Supplementary Fig. S5), suggesting that it can produce better guide trees for progressive multiple alignments, whereas the TN divergence based only on sequence shows the weakest correlations.

We then developed the new MSA program PC_ali that produces PAs that target PC_sim, either *de novo* or by modifying an input MSA. Next, PC_ali obtains a starting MSA from the sets of the maximal cliques of the graph of the PAs. Finally, PC_ali refines this starting MSA through iterative steps of progressive multiple alignments that adopt the guide trees based on PC_div and the similarity measure based on PC_sim, and it selects the MSA with largest PC_sim score.

We assessed the resulting MSAs on the Balibase database of structure-curated MSAs (Thompson *et al.* 1999). PC_ali achieves higher or not significantly different scores with respect to all other methods for the hybrid score PC_sim, the secondary structure similarity score, the CO, the TM-score, the aligned fraction, the similarity with the Balibase reference MSA, and the average similarity with other alignments (excluding those modified targeting PC_sim). It achieves higher sequence identity than other structure aligner (Mammoth, TM-align, or Balibase), which supports its suitability for evolutionary studies, but lower than sequence aligners such as MAFFT, T-coffee, and Muscle.

When PC_ali takes an MSA as input, it improves or mantains almost all scores of the input MSA. In particular, when the input is a structural MSA built by Mammoth or TM-align, PC_ali improves the sequence similarity, the aligned fraction, the structure similarities, and the similarity with other alignments (Supplementary Fig. S7), suggesting that it overcomes the limitations of these structural aligners that sometimes produces low sequence identity and short alignments. The improvement is very remarkable with TM-align, for which the above limitations are more pronounced, and it can be observed also for Balibase, which is considered a reference. Note that a recent study found that hybrid sequence–structure alignment methods perform worse than the program Mammoth (Carpentier and Chomilier 2019), while PC_ali largely improves upon Mammoth results.

When the input is an MSA based on sequence, PC_ali improves all structural scores, the similarity with other MSAs and with the reference MSAs of Balibase, it improves or maintains the aligned fraction except with respect to Muscle, and it worsens the sequence identity, except with respect to Clustal and Decipher (see Supplementary Fig. S7). However, the lower structure similarity scores and MSA similarity scores achieved by these methods make us suspect that the higher sequence score of some of them is due to overfitting.

Importantly, the MSAs constructed by PC_ali either *de novo* or starting from very different input MSAs have very similar (Fig. 4), showing that PC_ali is a robust method that produces high-quality alignments.

## Supplementary Material

btad630_Supplementary_Data

## Data Availability

The source codes needed for compiling and running PC_ali are freely available at https://github.com/ugobas/PC_ali. The results of the analysis of the Balibase multiple alignments and the superfamilies are available upon request to the corresponding author.
