# Supplementary material for: PC_ali: a tool for improved multiple alignments and evolutionary inference based on a hybrid protein sequence and structure similarity score
Source: Bioinformatics. 2023 Oct 17;39(11):btad630. doi: 10.1093/bioinformatics/btad630 (PMC10628387; doi:10.1093/bioinformatics/btad630)
Supplement: btad630_Supplementary_Data [file btad630_supplementary_data.zip › SuppMat_PC_sim.pdf]

Supplementary Material for the paper: PC_ali: A tool for multiple alignments and evolutionary inference based on a hybrid protein sequence and structure similarity score

Oscar Piette[(1)], David Abia[(1)] and Ugo Bastolla[(1,2)]
[(1)] Centro de Biologia Molecular "Severo Ochoa"
CSIC-UAM Cantoblanco, 28049 Madrid, Spain
[(2)] E-mail: ubastolla@cbm.csic.es

August 1, 2023

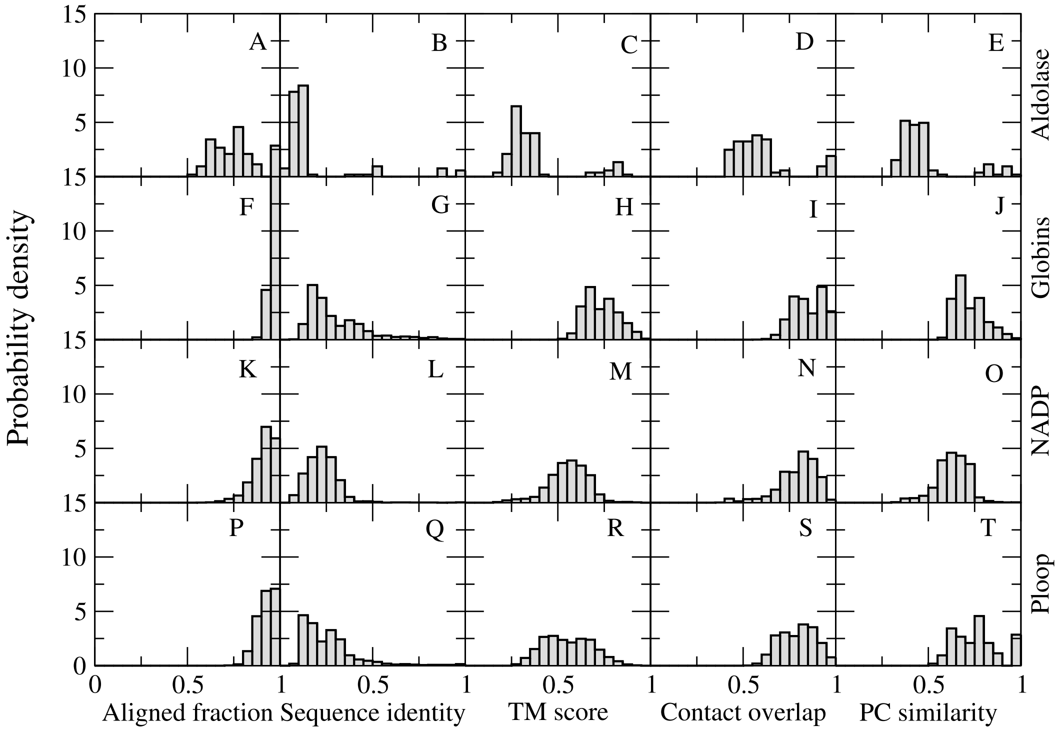

Figure 1: Distribution of the aligned fraction (1st column), Sequence identity (2nd column), TM score (3rd col), Contact overlap (4th col) and PC similarity (5th col) in the superfamilies Aldolase (110 pairs, plots A-E), Globins (2490 pairs, plots F-J), NADP (4191 pairs, plots K-O) and Ploop (2633 pairs, plots P-T). One can see that the four superfamilies span a very broad range of sequence identity.

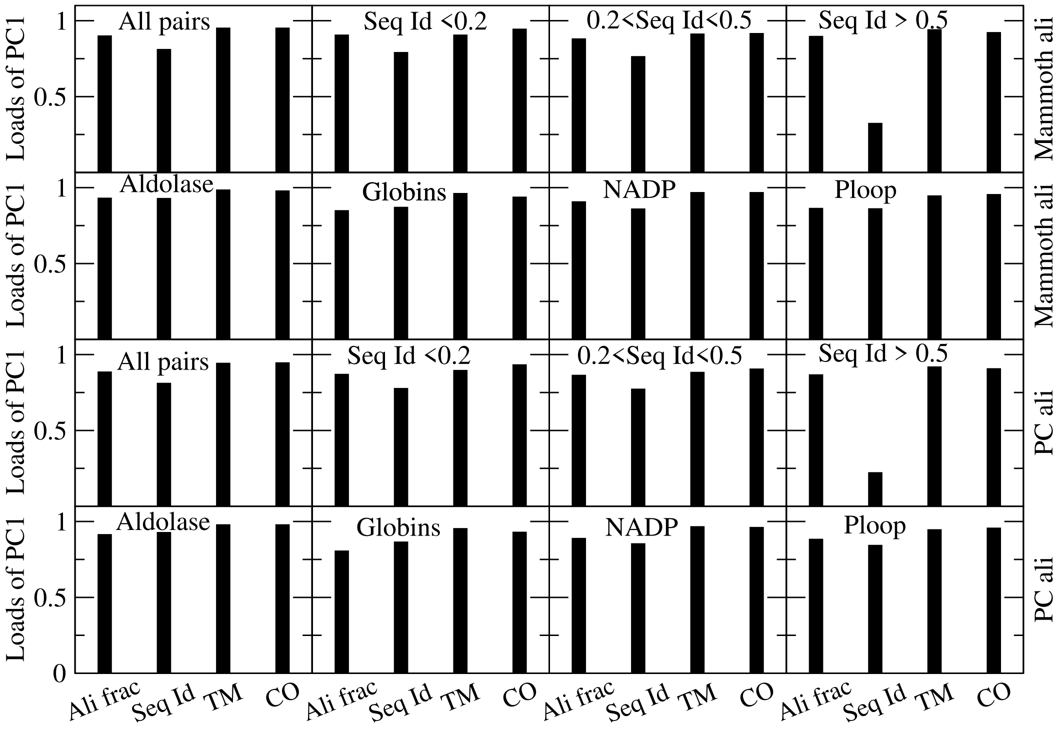

Figure 2: Loads of the first principal component of protein similarity measures (PC_sim) computed using all data (top left), pairs with low, intermediate and high sequence identity (top row) and pairs belonging to each of the four superfamilies (second row). The first two rows are obtained with the original Mammoth alignments, and the third and fourth are obtained with PC corrected Mammoth alignments. One can see that the PC loads are relatively robust, except that the contribution of sequence identity decreases at high sequence identity (5% of the pairs) due to its low correlation with aligned fraction and structure similarity measures, possibly because of the presence of conformational changes in the data set.

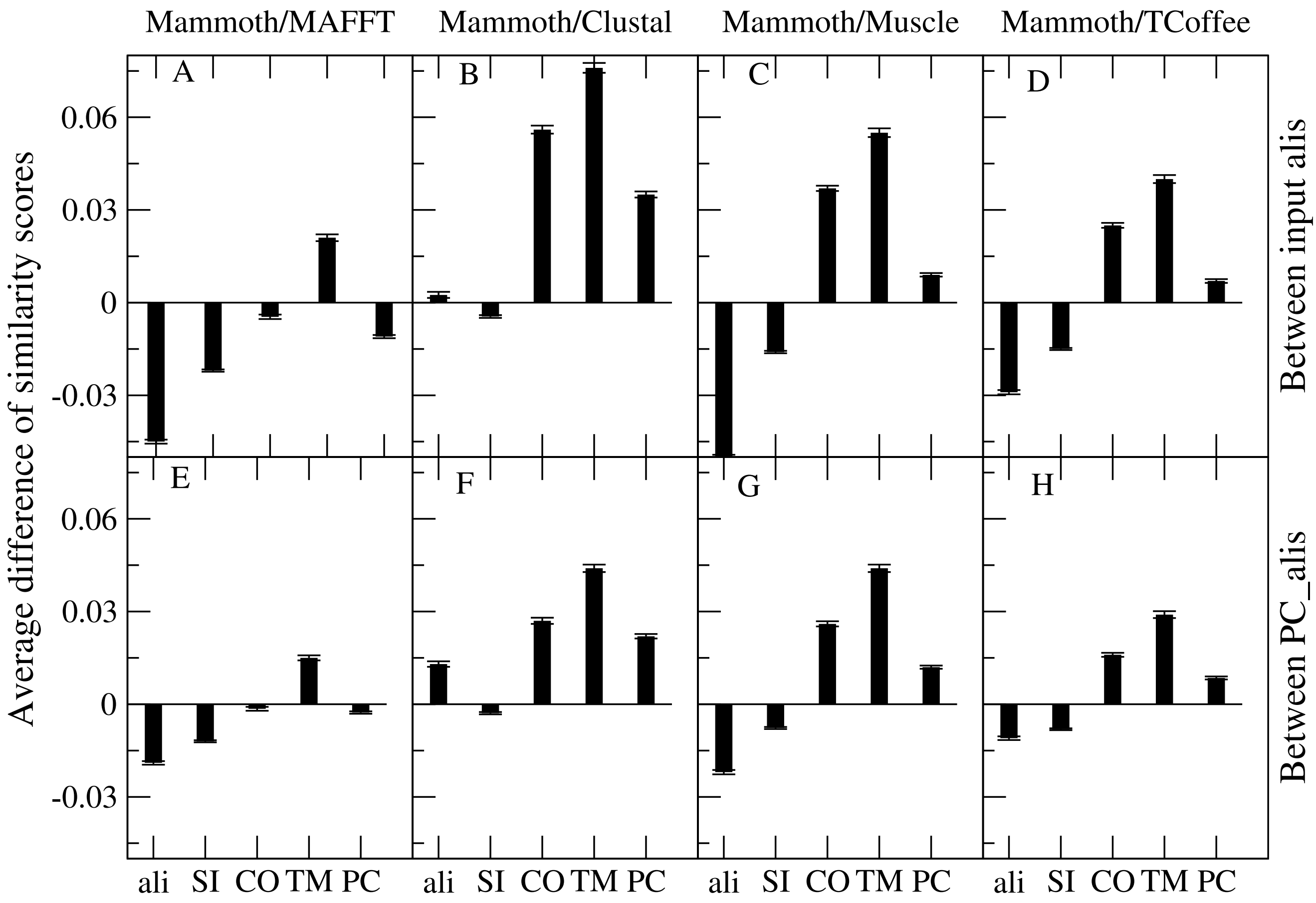

Figure 3: Influence of the input alignment on the similarity scores of modified pairwise alignments. Mammoth as input ali is compared to MAFFT (A,E), Clustal (B,F), Muscle (C,G) and Tcoffee (D,H). On the top line (A-D) the input alignments itself are compared, while on the bottom line (E-H) the PC_ali derived from them are compared. The error bars represent the standard error of the mean difference. One can see that the PC-modified alignments vary less than the corresponding input alignments (compare the top and bottom line). Note that MAFFT provides CO scores and PC scores higher than the structure aligner Mammoth, but the difference decreases after applying the PC correction.

Table 1: Loads of the four similarity measures for the computation of the first principal component PC_sim from different input alignments.

Program	Ali	SI	CO	TM
Muscle	0.71	0.81	0.94	0.91
Clustal	0.90	0.80	0.96	0.97
T-Coffee	0.84	0.81	0.95	0.93
MAFFT	0.84	0.79	0.95	0.95
Mammoth	0.90	0.81	0.96	0.96

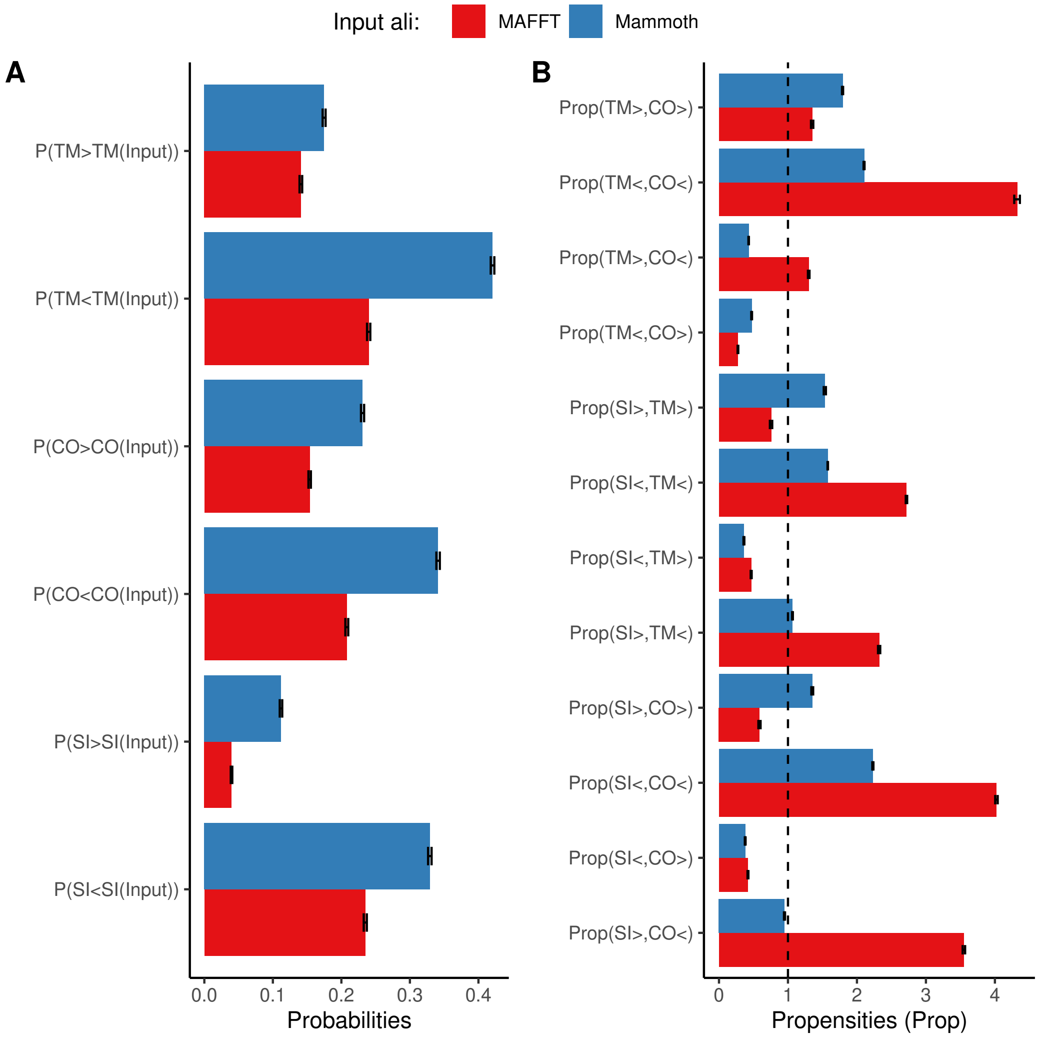

Figure 4: Performances of the SS_ali alignment modified based on secondary structure. SS_ali moves the gaps contained inside any secondary structure element (SSE) towards the closest end of the SSE, motivated by the idea that gaps inside a SSE might happen in evolution, so that the sequence alignment correctly infers homology, but the structure reorganizes so that the structural correspondence is different from the one dictated by the sequence alignment. If SS_ali is capturing such events, we expect that the SI tends to decrease when structure similarity scores TM and CO increase. Panel A represents the percentage of protein pairs for which any similarity increases or decreases with respect to the starting alignment, and it shows that in most cases SS_ali decreases the similarity scores SI, TM and CO, both with respect of the sequence aligner MAFFT and with respect to the structure aligner Mammoth. Panel B represents the propensities of all types of changes of similarity scores to occur together. It shows that increases of SI tend to be associated with increases of the structural scores TM and CO, and decreases of SI tend to be associated with decreases of the structural scores. This association is found both for starting alignments obtained with MAFFT and with Mammoth, and it suggests that SS_ali is either correcting alignment errors through the use of secondary structure information or introducing mistakes instead of dealing with genuine cases of indels inside SSE that motivated it.

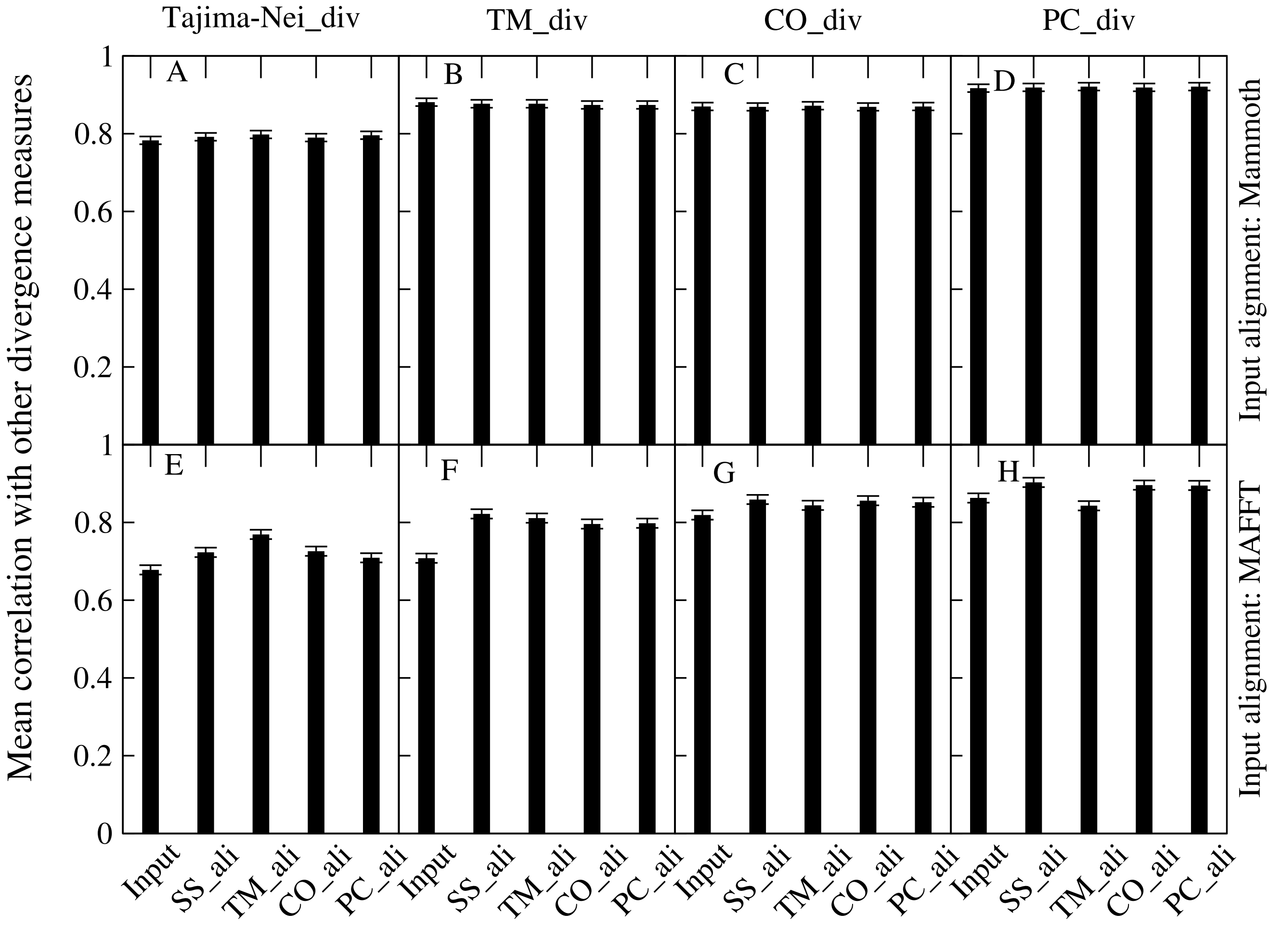

Figure 5: Correlation coefficients between different divergence measures for different types of modified pairwise alignments. In the top row the input alignment is Mammoth, in the bottom row it is Balibase. For all modified alignments and input alignments the highest correlations are attained with PC_div (D,H), and the lowest ones with the purely sequence-based Tajima-Nei divergence (A,E). Error bars represent standard deviation.

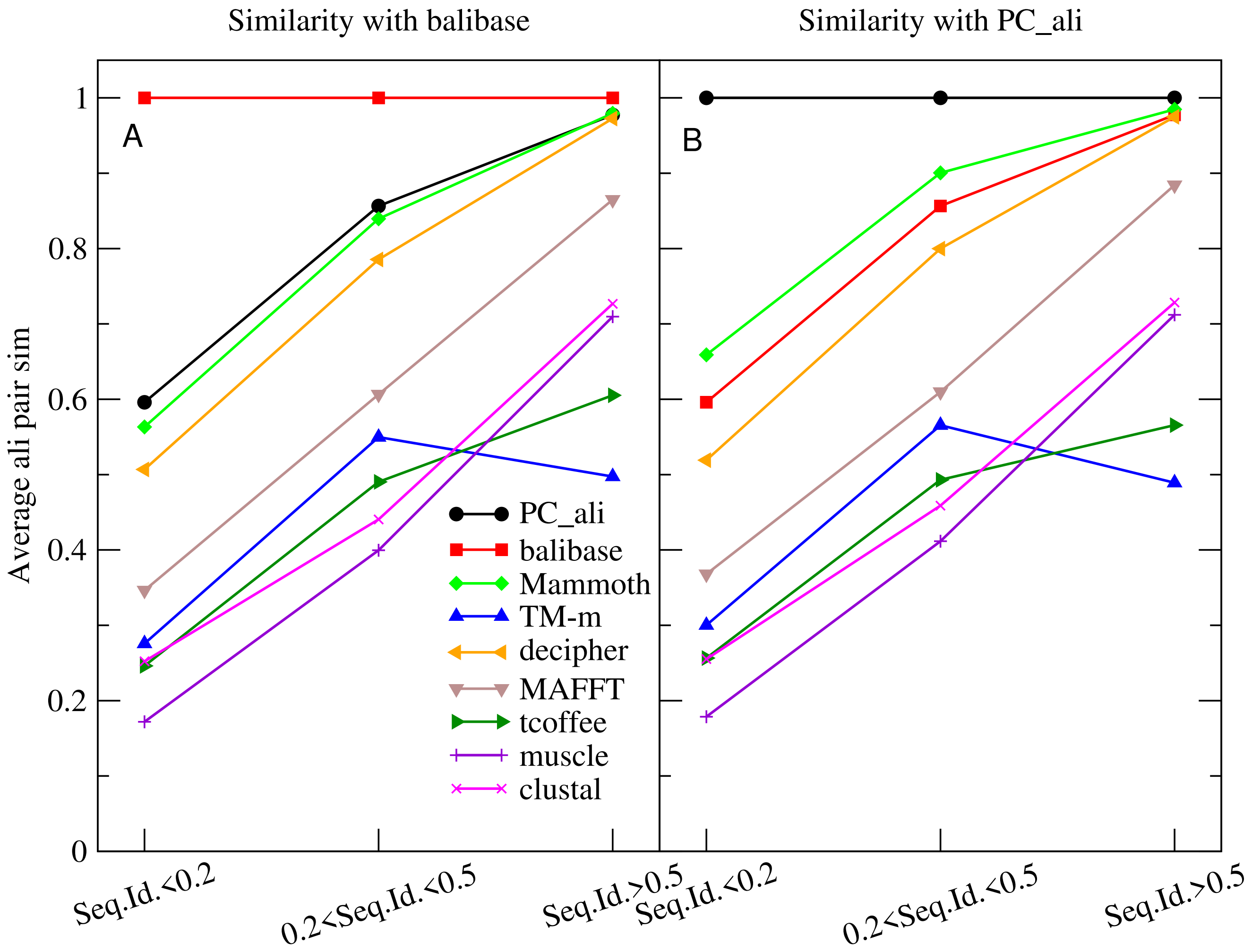

Figure 6: Influence of sequence identity (horizontal axis) on the similarity of the nine different MSAs with Balibase (plot A) and PC_ali (plot B). For difficult alignments with Seq.Id.<0.2, PC_ali is the MSA that most resembles the reference Balibase, followed by Mammoth and Decipher (plot A), and Mammoth is the MSA that most resembles PC_ali, followed by Balibase and Decipher (plot B). MAFFT is the best of the remaining methods, even higher than TM-align, and Muscle is the worst one.

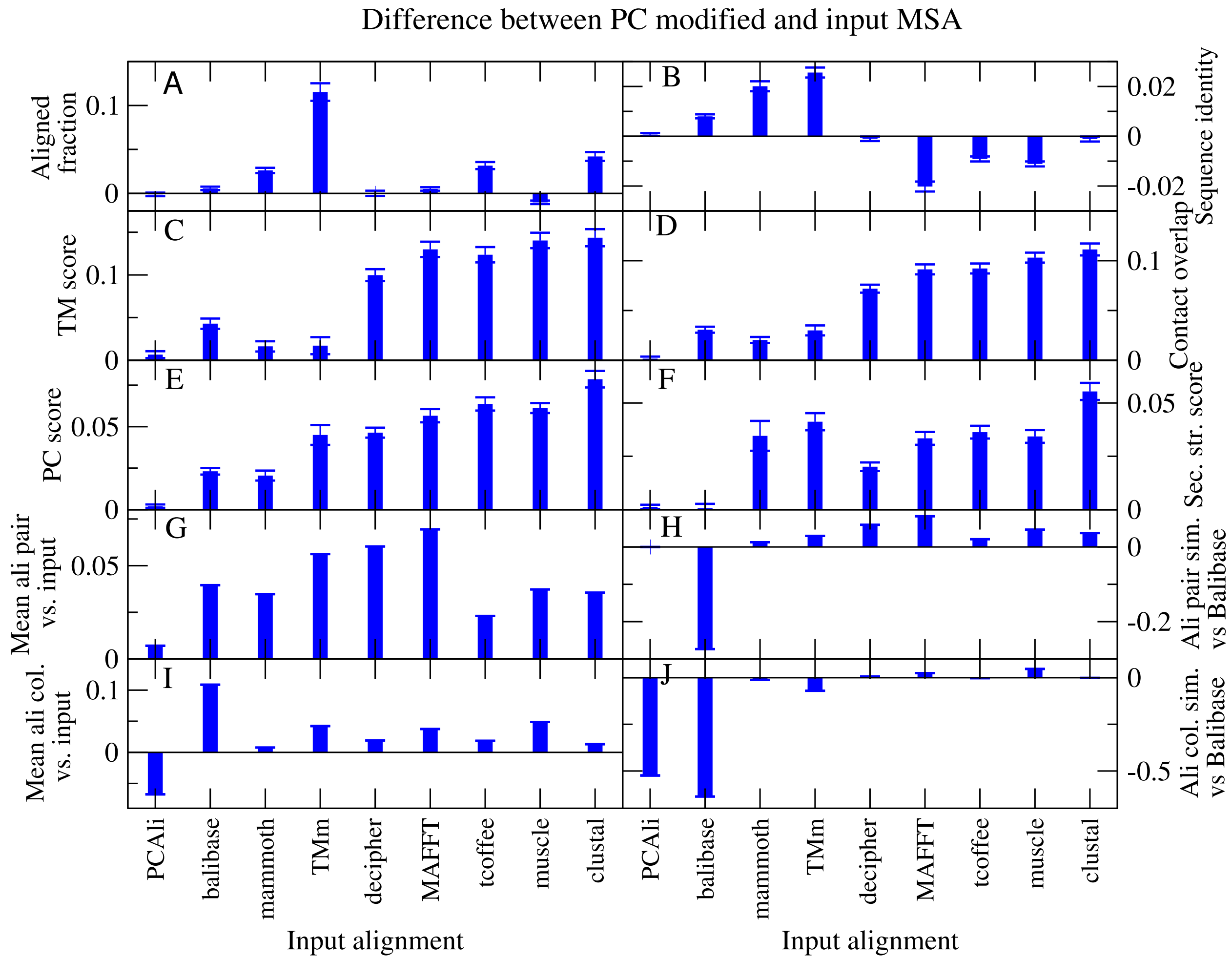

Figure 7: Score difference between the PC_ali modified MSA and the corresponding input MSA. The error bars represent the standard error of the mean and allow to visually assess the significance. A: Aligned fraction. B: Sequence identity. C: TM score. D: Contact overlap. E: PC_sim. F: Secondary structure identity. G, I: Average similarity with all other input MSAs, excluding the PC_ali modified MSAs (G: sum of pairs, I: column overlap). H, J: Sum of pairs similarity with the Balibase reference MSA. When the input MSA is a structural MSA built by Mammoth or TM-align, PC_ali improves the sequence similarity, the aligned fraction, the structure similarities and the similarity with other alignments, suggesting that it overcomes the limitations of these structural aligners that sometimes produces low sequence identity and short alignments. The improvement is very remarkable with TM-align, for which the above limitations are more pronounced, and it can be observed also for Balibase, which is considered a reference. When the input is an MSA based on sequence (Decipher, MAFFT, tcoffee, Muscle or Clustal), PC_ali improves all structural scores, the similarity with other MSAs and with the reference MSAs of Balibase, it improves or maintains the aligned fraction except with respect to Muscle, and it worsens the sequence identity, except with respect to Clustal and Decipher. However, the lower structure similarity scores achieved by these methods make us suspect that the higher sequence score of some of them is due to overfitting.
